# A Statistical Method to Base Nutrient Recommendations on Meta-Analysis of Intake and Health-Related Status Biomarkers

**DOI:** 10.1371/journal.pone.0093171

**Published:** 2014-03-28

**Authors:** Hilko van der Voet, Waldo J. de Boer, Olga W. Souverein, Esmée L. Doets, Pieter van 't Veer

**Affiliations:** 1 Biometris, Wageningen University and Research Centre, Wageningen, Netherlands,; 2 Division of Human Nutrition, Wageningen University, Wageningen, Netherlands; University College Dublin, Ireland

## Abstract

Nutrient recommendations in use today are often derived from relatively old data of few studies with few individuals. However, for many nutrients, including vitamin B-12, extensive data have now become available from both observational studies and randomized controlled trials, addressing the relation between intake and health-related status biomarkers. The purpose of this article is to provide new methodology for dietary planning based on dose-response data and meta-analysis. The methodology builds on existing work, and is consistent with current methodology and measurement error models for dietary assessment. The detailed purposes of this paper are twofold. Firstly, to define a Population Nutrient Level (PNL) for dietary planning in groups. Secondly, to show how data from different sources can be combined in an extended meta-analysis of intake-status datasets for estimating PNL as well as other nutrient intake values, such as the Average Nutrient Requirement (ANR) and the Individual Nutrient Level (INL). For this, a computational method is presented for comparing a bivariate lognormal distribution to a health criterion value. Procedures to meta-analyse available data in different ways are described. Example calculations on vitamin B-12 requirements were made for four models, assuming different ways of estimating the dose-response relation, and different values of the health criterion. Resulting estimates of ANRs and less so for INLs were found to be sensitive to model assumptions, whereas estimates of PNLs were much less sensitive to these assumptions as they were closer to the average nutrient intake in the available data.

## Introduction

Nutrient intake values (NIVs) have been introduced for assessment of an existing dietary situation or for planning a future situation [1], either for an individual or for a population. The focus of this paper is estimating NIVs for populations based on the availability of a multitude of reported data on intake and/or health-related status. Our purpose is to provide new methodology for dietary planning based on dose-response data and meta-analysis. The methodology builds on existing work, and is consistent with current definitions of NIVs, current methodology, and measurement error models for dietary assessment.

Current recommendations for various micronutrients were found to vary about 2-fold due to variation in approach, chosen health criterion, evidence base and decisions made [2]. Typically, the number of available data was small and often old. For example, current vitamin B-12 recommendations in the European Community, the USA, Canada and the Nordic countries are mainly based on a study begun in 1948 on only 7 patients with pernicious anemia [3], with results from six other studies being cited as qualitative support for the primary study [4]. Notably, that study used haematological status for health characterization, and not vitamin B-12 biomarkers because the major portion of the data was obtained prior to the existence of suitable methods for measuring them. In contrast, today results of many studies relating vitamin B-12 intake to biomarkers are available. For example, a systematic review of studies on vitamin B-12 intake and biomarkers of vitamin B-12 status identified 37 randomized controlled trials and 19 observational datasets as valid data sources [5]. Whereas current recommendations [4] are still mainly based on a roughly estimated mean requirement (2 μg/day), it may be time for updated recommendations using information on variability between individuals as has become available from the multitude of more recent studies using biomarkers.

Thus one objective of this paper is to propose an approach which utilizes all available validated data on intakes and health-related biomarkers. This includes data from RCTs and observational studies, and intake assessments using questionnaires as well as repeated 24-hour recalls. The other objective is to define the Population Nutrient Level (PNL) for planning intake in populations, and to propose a method to calculate PNL for cases where health-related data (e.g. status biomarkers) can be included.

Proposed methods are illustrated with an example for vitamin B-12. It is not the purpose of this paper to provide an updated recommendation on this micronutrient, but only to suggest a potentially useful statistical approach for integrated analysis of intake and biomarker data from multiple studies.

## Methods

### Data

#### Vitamin B-12 Intake-Status (IS) data

A systematic review on vitamin B-12 intake and biomarker relations is described in Dullemeijer et al. [5]. That paper restricted the attention to estimating a regression coefficient by meta-analysis, and therefore excluded studies that reported only on intake or only on status in a population. In short, the systematic review using wide search terms in order not to miss potentially useful papers identified 5913 papers, 49 of which met all inclusion and exclusion criteria of the review. The references to all basic studies are reported in Dullemeijer et al. [5]. These papers described 37 two-armed RCT datasets and 19 observational datasets on the intake-status relation. We refer to these intake-status data from RCT and observational studies as IS_rct_ and IS_obs_, respectively.

#### Vitamin B-12 Repeated Intake (RI) data

In this paper we perform a vitamin B-12 intake assessment for the Dutch adult population using consumption data with two repeats of a 24-hour recall (24HR) for 2230 adults (18-69 y) from the Dutch National Food Consumption Survey 2010 [6]. Vitamin B-12 concentration data were taken from the Dutch Food Composition Tables [7] and were the same as used in a recently reported study [8].

#### Vitamin B-12 Repeated Status (RS) data

In a longitudinal study 22 healthy people were followed for one year and serum vitamin B-12 measurements were repeated four times for each person [9].

### Current methods

Methodology to assess or plan nutrient intakes was established in two reports of the Institute Of Medicine (IOM) in the US [10], [11], and has been summarized with examples [12]. In this paper we mainly use the harmonized terminology for NIVs at the international level [1]. The *Average Nutrient Requirement* (ANR) [1], also known as *Estimated Average Requirement* (EAR) [10] or *Average Requirement* (AR) [13], is the average or median requirement estimated from a statistical distribution of requirements for meeting a specific health criterion and for a particular age- and sex-specific group [1]. The term *population* will be used for such an age- and sex-specific group, but also for the entire group of all ages and both sexes when appropriate. The ANR in combination with the *variation in nutrient requirements* in a population, typically set as a coefficient of variation (CVNR) or a standard deviation (*SDNR*), can be used to derive an *Individual Nutrient Level* for p% of the population (INL_p_) [1], also known as *Recommended Dietary Allowance* (RDA) [10] or *Population Reference Intake* (PRI) [13]. Typically the percentage p might be 97.5% (sometimes rounded to 98%). Then, using *italic* script to indicate logarithmically transformed values (as is motivated later), *INL_97_*_.5_  =  *ANR* +*2•SDNR* is the recommended nutrient level for any healthy individual in this population, and the recommendation is meant to restrict the probability to 2.5% that an intake of INL_97.5_ does not meet the individual's requirement.

At the group level a calculation of NIVs will start with estimating the current *usual nutrient intake distribution* for the population for which the data are considered to be representative. Often this distribution is assumed to be normal, possibly after an appropriate data transformation, e.g. the logarithmic transformation. Usual intake distributions cannot be observed directly, but can be estimated from surveys with a small number of repeated observations for each individual, often 24-hour recalls [14]. Different statistical methods for estimation of the usual intake distribution exist and have been compared [15]–[19]. If normality at some appropriate scale is reasonable then the usual nutrient intake distribution can be summarized by the average nutrient intake (*ANI*) and the standard deviation of (usual) nutrient intake (*SDNI*). A method for assessing nutrient inadequacy in a population is the cut-point (or EAR cut-point) method [10], [20]–[22]. It simply consists of estimating the percentage of the usual nutrient intake distribution below ANR. For the cut-point method to be valid, several assumptions have to be fulfilled [10]: intakes and requirements are independent, the requirement distribution is symmetrical around *ANR*, and the variation in intakes is larger than the variation of requirements (*SDNI* > *SDNR*).

In line with this evaluation method, the IOM [11] also proposed a method for planning nutrient intake for groups, i.e. to plan for a median nutrient intake enough to exceed the Average Nutrient requirement (ANR) for 97.5% of the population. This can be achieved by calculating the Median of the Target Usual Nutrient Intake Distribution (*MTUNID*) as *ANR* + 2*SDNI*, where it is assumed that the Standard Deviation of Nutrient Intake (*SDNI*) remains the same in the future scenario.

### The population-based bivariate lognormal model for intake-status data

**Figure 1:**
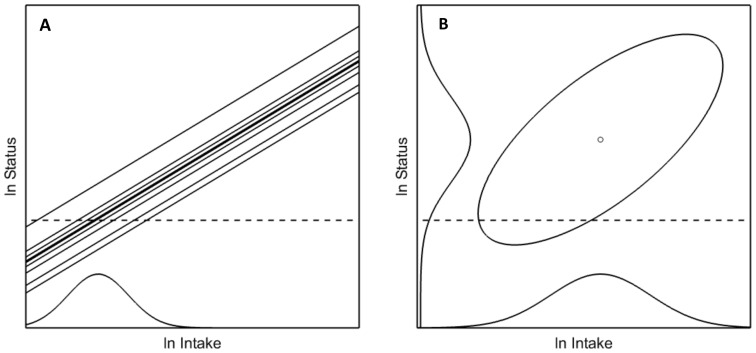
Ln status vs. ln intake. (A) Model of parallel individual regression lines defines the distribution of nutrient requirements. The health-related cut-off value for ln status is depicted by the horizontal dotted line. The intersection of the parallel dose-response lines with the cut-off value defines the requirements distribution, as shown along the ln Intake axis. (B) The intake-status model shows a bivariate normal distribution representing ln intake and ln status in a population of individuals. Marginal intake and status distributions are shown along the respective axes.

We define a general population-based model for the case that measurements are available on intakes and at least one health-related variable. For example, in relation to health problems due to an insufficient intake of vitamin B-12, measurements are available of a health related biomarker such as serum or plasma vitamin B-12. A limit value at such a scale determines whether an individual has sufficient health. In the example a cut-off of 150 pMol/L for plasma vitamin B-12 suggested in a WHO Consultation [23] is assumed to classify the individual's health as sufficient or insufficient. By definition, the intake at which there is 50% probability of meeting the cut-off value is the average nutrient requirement (ANR). Note that intake requirements are expressed on the intake scale. Usually no direct measurements of individual dose-response relations are available, so individual intake requirements are unobservable. In our model we assume that each individual has a dose-response relation linking intake to the health-related status variable. Variation in requirements is modelled by a family of parallel dose-response functions (see Figure 1A). These lines cross the horizontal line representing a fixed cut-off value. Note that the variation between the lines induces a variation in requirements. The intakes corresponding with the points where the dose-response lines cross the horizontal criterion line define the requirement distribution. ANR and INL_p_ are defined as the median and the p^th^ percentile of this distribution. Whereas INL_p_ is a recommendation for an individual, at the level of the population *p* % of the population would have sufficient intake if all individuals would consume exactly this amount of the nutrient, i.e. when dietary recommendation would remove all dietary variation in the population. This is obviously not what is expected to happen after a recommendation is given. We here assume the simplest model, i.e., that median intake will shift by a certain factor to a recommended level, but that the relative variation remains unaffected. For such applications, we define the *Population Nutrient Level* (PNL_p_) as the median of the target nutrient intake distribution, such that p % of this population will have sufficient intake.

Intake and health-related markers are often continuous variables bounded by zero. Distributions may be skewed if the variation in values is large relative to the mean value. A general approach for positive data is to apply a logarithmic transformation, and then apply modelling to the log transformed data. We use natural logarithms (with base number *e*, and denoted by ln), but any other choice of base number would give equivalent results.

The joint distribution of intake and health in a population of interest is modelled by a bivariate stochastic model (Figure 1B). Variability between persons exists for both intakes and health outcomes. To characterise the bivariate normal distribution for *I* =  ln(true intake) and *S* =  ln(true status) we choose the following 5 parameters: average nutrient intake (*ANI*), average nutrient status (*ANS*), SD of nutrient intake (*SDNI*), SD of nutrient status (*SDNS*), and the regression coefficient 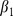 of the relation to predict *S* from *I*. We use *italic font* (e.g. *ANI*) for quantities at the ln scale, and regular font (e.g. ANI) for the back-transformed quantity. Note that the A of ‘Average’ therefore relates to a geometric rather than a arithmetic mean, as is already customary use in e.g. ANR or EAR.

The relation between the model for requirements (Figure 1A) and the model for intake-health relation (Figure 1B) is that a bivariate normal distribution implies a linear regression line when predicting one of the variables based on the other. For error-free observations this relation is
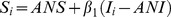
(1)


with subscript *i* indicating any individual person in the population of interest.

**Figure 2:**
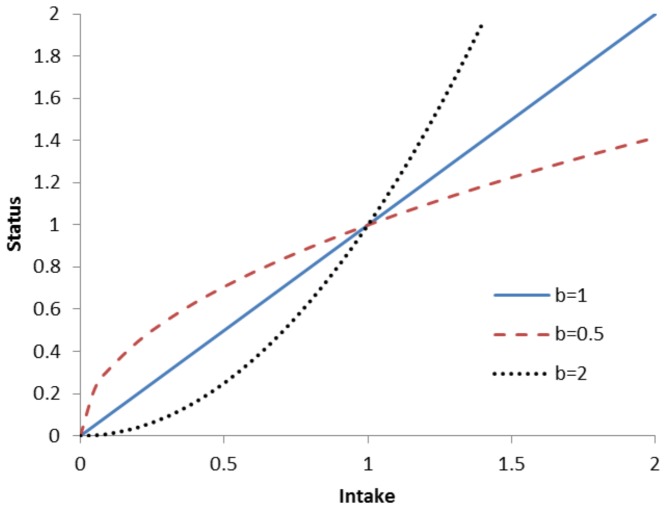
Hypothetical Intake-Status relations which can all be represented by linear functions on the ln-ln scales.

Linearity is a strong assumption, but its use can be motivated by observing that the form of true relationships between intake and status are often masked by large measurement errors. A linear relation is then the common practical first-order approximation. In addition, a linear relation between intake I and status S on the ln-ln scale, ln(S)  = a+b ln(I), corresponds to a power function on the original scale, S = e^a^ e^b ln(I)^  =  k I^b^, with k being a constant multiplier. This is a concave function for b<1 and a convex function for b>1. Therefore using a simple linear model on the ln-ln scale is compatible with specific curvilinear functions on the original scale (Figure 2). Specifically, curves showing some sort of saturation (concave curves) can be approximated by ln-ln linear functions with b<1. We note that the linearity assumption is also used in the IOM report on dietary reference intakes [11]. Nevertheless, the assumption should always be critically investigated and predictions extrapolated outside the domain of the original data should be taken only as qualitative indications.

It is assumed that true regression lines differ between individuals because of variation in individual requirements. On top of this measurement errors in *I* and *S* exist and only error-prone measurements *x* of *I* and *y* of *S* are available. Under a model of parallel regression lines for random individuals in the population and absence of a general bias in the observed intake the following equations can be derived for nutrient intake levels (see Appendix S1 for details of the derivation):
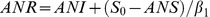
(2 = A.5)




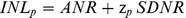
(3 = A.6)




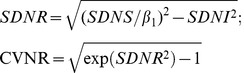
(4 = A.8)




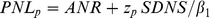
(5 = A.10)


*ANR* is therefore the intersection of the horizontal line *y* =  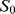
with the line through the point (*ANI*, *ANS*) with slope 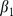. *INL_p_* is *ANR* plus an appropriate multiple of the requirements standard deviation *SDNR*. *PNL_p_* is defined as the intake level where p % of the *S* distribution is above *S_0_*.

For comparison with the MTUNID advocated in the IOM report [11], the PNL can be rewritten using the familiar relation between regression and correlation in a bivariate normal distribution (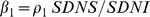). This leads to
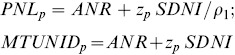
(6)


*MTUNID_p_* is therefore the same as *PNL_p_* if the correlation 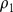 between true intake and true status equals 1, i.e. if the variation in status at a given intake level is negligible, or, equivalently, if the parallel lines in Figure 1A are very close together. This is the equivalent of the assumption behind the cut-point method that the variation in requirements should be small compared to the variation in intake.

### Estimating the parameters of the model

The NIVs (ANR, INL and PNL) can be derived from the five parameters (*ANI*, *ANS*, *SDNI*, *SDNS* and 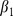) of the bivariate stochastic model plus the health-defining status level 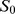. In this paper we estimate *ANI*, *ANS* and *SDNS* from a meta-analysis of observational data (IS_obs_ data). For the latter parameter we also use a published study on repeated status measurements to correct the SD of the observed status measurements (*SDy*) to *SDNS* (RS data). Note that in our basic model we do not estimate the remaining parameters *SDNI* and 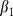 from the observational data because of unknown measurement error which is expected to inflate the observed intake variation and attenuate the observed slope. Rather, we rely on repeated 24HR data for *SDNI* (RI data), and, in our basic model, on RCT data for 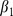 (IS_rct_ data).

The procedure to base nutrient recommendations on intake and health-related measurements proposed in this paper consists of five steps (Table 1). Some of these steps have already been described in other papers, as indicated in the table.

**Table 1:** Steps in the extended meta-analysis procedure to base nutrient recommendations on intake and health-related measurements

Step	Description	Ref
1	Select valid studies on intake, status, and their relation;	5
	both RCTs and observational	this paper
2	Transform summary statistics for use to estimate parameters of the bivariate log-normal model	24
3	Perform (meta-)analysis to derive regression coefficients, separately for RCTs (*b_rct_*) and observational IS studies (*b_obs_*)	5
4	Depending on assumptions and data checks, estimate the bivariate lognormal model parameters *ANI*, *ANS*, *SDNI*, *SDNS* and 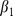	this paper
5	Based on an external health-related cut-off value 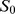 derive estimates of ANR (EAR), INL (RDA), PNL	this paper

**Step 1** involves the definition of a search strategy to find possibly useful reports of studies on intake and/or status, typically by database searches. It also involves setting criteria for inclusion/exclusion, procedures for data extraction and data synthesis, and assessments of the validity of included studies, e.g. by assessing the adequacy of random assignment and blinding in RCTs and by assessing the possible influence of confounders such as mean age in a meta-analysis of observational data.

#### Intake-Status (IS) data

For the example of vitamin B-12 this step has been described extensively in Dullemeijer et al. [5].

#### Repeated Intake (RI) data

In the analysis of the RI data we assume the simple model
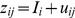
(7)and variance components for ln intake were estimated for between-individual (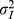) and within-individual (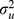) variation using the BBN method [17] in the program MCRA (available at https://mcra.rivm.nl). According to this model *SDNI* is the square root of 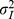.

Intake-related bias was found to be present in the OPEN study [25] not only for frequency questionnaire data, but also for repeated 24-hour food recall data on energy and protein. In a joint analysis of biomarker, repeated frequency questionnaire and repeated recall data, the repeated food recall data were modelled as
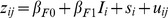
(8)


The slope in the regression of 24HR-reported on true intake (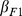) was reported to be between 0.46 and 0.70 for energy and protein in males and females ([25], Table 2). Note that *I_i_* and *s_i_* cannot be distinguished in a model assuming no intake-related bias (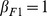), and the *SDNI* estimate from model 7 would represent both *I_i_* and *s_i_*. For vitamin B-12 not enough data are available to estimate model 8. In the presence of intake-related bias in the 24-hour recall data of similar magnitude as for energy and protein the *SDNI* estimate from model 12 can be corrected by multiplying with a factor 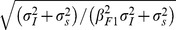, using the estimates derived from model 8. For energy and protein in males and females in the OPEN study these factors work out to be between 1.16 and 1.27.

**Table 2:** Models for estimation of the regression coefficient 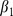
in 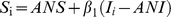.

Model	Estimate of 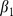	Assumption
A (RCT-based)	Meta-analysis of RCT Intake-Status data	Linear dose response in
	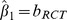	RCTs (on the ln-ln scale)
B (obs-based)	Meta-analysis of observational Intake-Status data + de-attenuation	No intake-related bias,
	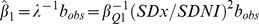	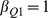
C (CVNR 20%)	Observed Intake and Status variation + de-attenuation	Traditional value for
	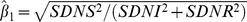	variation in requirements, CVNR = 20%
D (max slope)	Observed Intake and Status variation + minimal de-attenuation	No variation in
	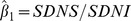	requirements

#### Repeated Status (RS) data

For serum vitamin B-12 McKinley et al. [9] reported a reliability coefficient *RC* (coefficient of variation (CV) between individuals divided by total CV) of 0.97. This value was used to correct the observed variation in nutrient status to 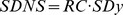.

We assume that after completion of Step 1 all remaining data are valid for use in meta-analysis. Here, validity includes absence of serious general bias, but not necessarily absence of intake-related bias or random measurement error.

In **Step 2** the available data are re-parameterised to fit the bivariate log-normal model. Literature reports mostly do not report original data, but only a variety of summary statistics. For example, reported univariate statistics may be means, medians, standard deviations, inter-quartile ranges, ranges, confidence intervals, either on the original scale or a transformed scale. Bivariate statistics may be Pearson or rank correlation coefficients or regression coefficients based on original or transformed variables. Souverein et al. [24] have described transformations that can be used to transform such summary statistics to basic single-study estimates of parameters of the bivariate log-normal distribution. For example, means *(m)* and SDs (*s*) at the ln scale can be derived from medians (MED) and CVs on the original scale by 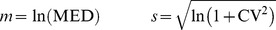
(9)


In **Step 3** a meta-analysis of the RCT and observational data is performed to derive estimates of the intake-status relation regression coefficient. The random-effects meta-analysis can be performed in various statistical programs, using the moments method of DerSimonian and Laird [26] or Residual Maximum Likelihood (REML) [27] to estimate the between-study variance. Without measurement errors all data could be combined to derive an overall estimate. However, measurement error in intake will attenuate the estimate from observational data, but not in most RCT data where the high dose group has a fixed known level.

In **Step 4** the crucial task is estimating the true slope 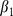, which can be difficult in practice. At high intakes one may expect saturation of the response, therefore the question can be raised whether a simple (ln-ln) linear model can be used for RCT data with high doses. Observational data on the intake-status relation will show attenuation of the slope estimate. The attenuation factor is defined as the covariance between true and observed intakes divided by the variance of the observed intakes, and can be expressed, using elementary statistical relations between covariance, correlation and regression coefficient in a bivariate normal distribution, as:
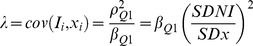
(10)


In this paper we explore a possible range of values for 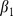 based on different assumptions (see Table 2). In the basic model (A) a meta-analysis of RCTs is used to estimate 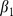. This estimate is considered as a practical minimum, because saturation at high intake levels in RCT data would attenuate the slope, implying that it would be steeper at the ‘natural’ lower levels of intake.

In other models we avoid use of the RCT data. In model B the observational slope is de-attenuated based on equation 10. The attenuation factor is estimated from a comparison of intake variation in the observational IS and repeated 24HR datasets, with the additional assumption that there is no intake-related bias in the observational intake-status data. In models C and D an estimate of 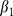 is based on equation A.7 plus the assumption that the variation is nutrient requirements is known to be 20% [28] or 0%. The latter choice leads to the maximum possible value for 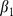.

In **Step 5** the estimated error-corrected intake-status distribution is used to derive nutrient intake values, using equations 2, 3, 5 and 6.

## Results

### Steps 1-3. Study selection, summary statistics transformation and meta-analysis of regression coefficients

The results of the Intake-Status studies have been described in previous publications [5], [24]. After transforming the published summary statistics to a common scale, a random-effects meta-analysis was performed, using the method of DerSimonian and Laird [26] to estimate the between-study variance. This procedure resulted in estimates *b_rct_*  = 0.17 (95% CI 0.15–0.20) and *b_obs_*  = 0.10 (95% CI 0.06–0.14). The observed attenuation factor is therefore 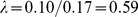.

### Step 4. Estimate the model parameters ANI, ANS, SDNI, SDNS and 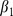


The Repeated Intake (2×24HR) data showed intake of vitamin B-12 on both survey days for 2190 of the 2240 individuals (98.2%), and on one survey day for 38 individuals (1.7%). Restricting attention to the positive intakes the between and within-individual variances for ln(intake) were estimated to be 0.2037 and 0.6176, respectively. The median intake was 3.38 μg/day, and the 2.5^th^ and 97.5^th^ percentiles of the estimated long-term intake distribution were 1.3 and 7.9 μg/day.

Estimates of the 5 parameters of the stochastic Intake-Status model are shown in Table 3 (univariate statistics) and Table 4 (the regression coefficient for different models). The estimated regression coefficients vary by more than a factor 5, from 0.172 for the RCT-based model A to 0.926 for the max slope model D. When RCT data are used (model A) this implies, from equation 15, a positive intake-related bias characterised by 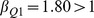. In fact, intake-related variation then explains 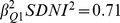, which is more than the total variance observed in *x* (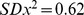), and therefore residual variation (the term *d_i_* in model 2b) must play a negligible role. Under model A the correlation between true intake and true status is low (0.19), and the coefficient of variation for the nutrient requirements is very large (1736%).

**Table 3:** Estimated means and standard deviations.

Data used	Ln scale		Original scale	
Intake-Status observational data (IS_obs_)	*ANI*	1.40	ANI	4.05 μg/day
Intake-Status observational data (IS_obs_)	*ANS*	5.69	ANS	296 pMol/L
Repeated intake data from 24 hour recall	*SDNI*	0.451	CVNI	47.5%
IS_obs_ and correction from repeated status data	*SDNS*	0.418	CVNS	43.7%

**Table 4:** Vitamin B-12 example.

Model	Data used for 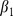 1	Slope 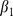 (cf. Table 2)	Correlation 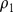 2	Attenuation factor 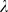 3	Intake-related bias 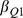 4	CVNR (%)^5^
A (RCT-based)	IS_rct_	**0.172**	0.19	0.589	1.80 (100%)	1736
B (obs-based)	IS_obs_, RI	0.310	0.33	0.326	**1 (0%)**	201
C (CVNR 20%)	IS_obs_, RI, RS	0.848	0.92	0.119	0.343 (12%)	**20**
D (max slope)	IS_obs_, RI, RS	0.926	1.00	0.109	0.335 (13%)	**0**

1 IS_rct_: Intake-Status RCT data; IS_obs_: Intake-Status observational data; RI: Repeated Intake data; RS: Repeated Status data.

2 Correlation between *I* and *S* calculated as 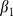 multiplied by *SDNI*/*SDNS*.

3 Attenuation factor defined as ratio of *b_obs_* to 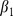.

4 Calculated from equation 10. The percentage in parentheses indicates how much of the total variance of differences between observed and true log-intakes (*x_i_* –*I_i_*) is explained by intake-related bias.

5 Coefficient of Variation of Nutrient Requirements, calculated from equation 4.

Estimates of association parameters (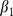,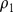) and related statistics. Inputs according to the chosen model are shown in bold.

In model C the CVNR is set to a more traditional value of 20%. In that case the regression coefficient is estimated to be 0.848, and a negative intake-related bias is found characterised by 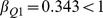, i.e. more similar the values for energy and protein found in the OPEN study [25], which ranged between 0.24 and 0.83.

### Step 5. Estimate Nutrient Intake Values

**Figure 3:**
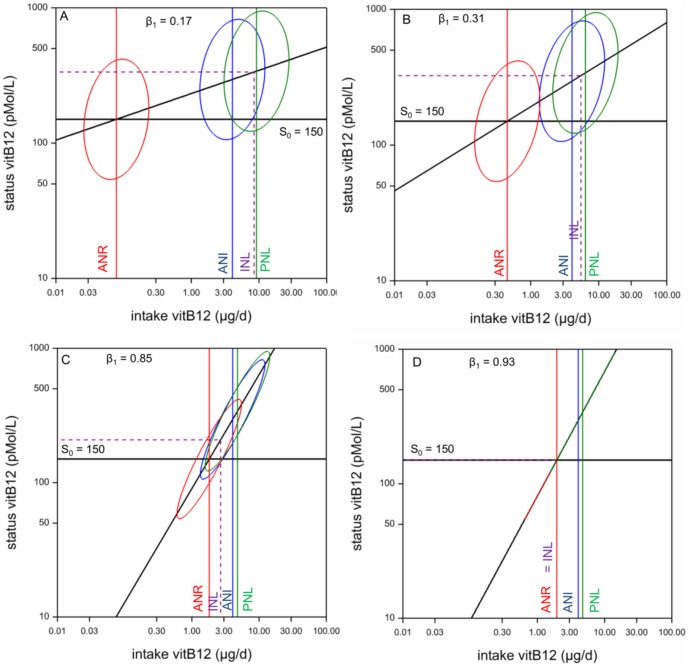
Deriving nutrient intake values for vitamin B-12 from the bivariate lognormal model. Four models (A–D, see Table 2) of using the dose-response relation (sloping line) and estimated current distribution around the Average Nutrient Intake ANI (blue ellipse) for estimating the Average Nutrient Requirement ANR (50% below line S_0_ = 150, red ellipse), the Individual Nutrient Level INL (2.5% of requirements distribution above purple vertical line) and the Population Nutrient Level PNL (2.5% below line S_0_ = 150, green ellipse).

The parameter estimates made in Step 4 can be combined with a health-related cut-off value 150 pMol/L to calculate the Nutrient Intake Values (Table 5 and Figure 3). Based on the data used, the adequacy of the nutrient status in the population is estimated to be higher than 50%, but lower than 97.5%, therefore in the order A-B-C-D the steeper slopes of the dose-response line lead to increasing values for ANR, but decreasing values for PNL_97.5_. The values of INL_97.5_ show an even stronger decreasing series because of the enormous decrease in CVNR from A to D (in the last model CVNR = 0 and therefore INL_97.5_ = ANR). Finally, the IOM-proposed MTUNID_97.5_ follows the same increasing pattern as ANR because it is just multiplied by a factor which is equal for the four models (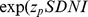) = 2.5). For information, the ratio of the NIVs according to A and D has been added in the last row of Table 5.

**Table 5:** Estimated Nutrient Intake Values for four models (A–D) based on health-related cut-off value 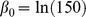.

Model	ANR (EAR)	INL_97.5_ (RDA)	PNL_97.5_	MTUNID_97.5_
	(μg/d)	(μg/d)	(μg/d)	(μg/d)
	(Eq. 5)	(Eq. 6)	(Eq. 10)	(Eq. 11b)
A (RCT-based)	0.078	8.5	9.2	0.19
B (obs-based)	0.45	5.5	6.4	1.1
C (CVNR 20%)	1.8	2.7	4.8	4.4
D (max slope)	1.9	1.9	4.7	4.7
ratio A/D	0.04	4	2	0.04

Recently, higher cut-off values 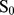 for plasma vitamin B-12 have been recommended [29], [30]. For illustration we show one example (200 instead of 150 pMol/L) in Table 6. Further calculations show that cut-off values of 258 or 300 pMol/L would increase the ANR estimate in model A to 1.8 or 4.4 μg/d and the PNL_97.5_ estimate to 216 or 518 μg/d, respectively. For model C ANR would be increased to 3.4 or 4.1 μg/d and the PNL_97.5_ estimate to 9.1 or 10.8 μg/d, respectively.

**Table 6:** Estimated Nutrient Intake Values for four models (A–D) based on health-related cut-off value 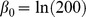.

Model	ANR (EAR)	INL_97.5_ (RDA)	PNL_97.5_	MTUNID_97.5_
	(μg/d)	(μg/d)	(μg/d)	(μg/d)
	(Eq. 5)	(Eq. 6)	(Eq. 10)	(Eq. 11b)
A (RCT-based)	0.42	45	49	1.0
B (obs-based)	1.1	14	16	2.8
C (CVNR 20%)	2.6	3.8	6.7	6.2
D (max slope)	2.7	2.7	6.4	6.4
ratio A/D	0.16	17	8	0.16

Considering the situation from the other side, the ANRs for vitamin B-12 proposed by IOM, European and other scientific advisory bodies range from 1.0 to 2.0 μg/d. Under the settings of model A this implies that implicitly the cut-off values for the status parameter range between 232 and 262 pMol/L, well above the cut-off of 150 pMol/L proposed by the WHO Consultation. Under model C this range would be between 90 and 163 pMol/L.

A sensitivity analysis was performed for the magnitude of *SDNI*. As explained above, *SDNI* could have been estimated too low because we fitted a model without intake-related bias to the 24HR data. In the sensitivity analysis (Table 7) we calculated the NIVs based on a 25% increased value for SDNI, which is a typical correction factor that would be appropriate if intake variation properties of vitamin B-12 intake would resemble those of energy and protein in the OPEN study.

**Table 7:** Estimated Nutrient Intake Values for four models (A–D) based on health-related cut-off value 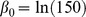 and a 25% increased *SDNI*.

Model	ANR (EAR)	INL_97.5_ (RDA)	PNL_97.5_	MTUNID_97.5_
	(μg/d)	(μg/d)	(μg/d)	(μg/d)
	(Eq. 5)	(Eq. 6)	(Eq. 10)	(Eq. 11b)
A (RCT-based)	0.078	8.1	9.2	0.24
B (obs-based)	0.13	7.1	8.2	0.40
C (CVNR 20%)	1.5	2.3	5.0	4.6
D (max slope)	1.6	1.6	4.9	4.9
ratio A/D	0.05	5	2	0.05

## Discussion

### General

We have outlined theory and methodology for deriving nutrient intake values such as ANR (EAR), INL (RDA) and the newly defined PNL by statistically combining results from epidemiologic studies, intervention trials and food consumption surveys. The main conclusion is that it is possible to derive NIVs using a larger body of evidence than is commonly done. The underlying model is consistent with current methods to evaluate and recommend nutrient intake for populations. The model requires a limit value for a health-related status variable, similar to other methods to derive NIVs.

The proposed Population Nutrient Level (PNL) is conceptually equal to the Median of the Target Usual Nutrient Intake Distribution (MTUNID) defined by IOM [11]. However, whereas the IOM methods assumes limited variation in intake requirements (10 to 20%), the proposed method starts from bivariate intake-status data, and considers variations in requirement to be a non-negligible source of the residual variation around the dose-response function.

Whereas dieticians may be most interested in INL for individual advice, public health policy-makers should set PNL to attain their goals. In other words, policy-makers should be concerned not only with a mean level of intake, but also with the variation in intake between individuals in a population. This is in line with the concepts behind the cut-point method for evaluating population nutrient intake [21] as well as the Target Usual Nutrient Intake Distribution proposed by IOM [11].

In general, the main strengths of the proposed model relative to the current methodology as exemplified in the IOM reports are the use of biomarker (status) data to estimate the variation in requirements, and NIVs estimated by combining information from multiple relevant datasets of different types (IS_rct_, IS_obs_, RI and RS data). A weakness of our model, that it shares with the IOM method, is the reliance on assuming a linear relation on the ln-ln scale. The fact that our model framework allows different models to be formulated (see the example models A–D in this paper), each based on partly different data, can be seen as either a strength or a weakness, depending on one's point of view.

Resulting estimates of ANRs and less so for INLs were found to be sensitive to models assumptions, whereas estimates of PNLs are much less sensitive to these assumptions as they are closer to the “bulk” of the available data.

### Data

An advantage of the model is the use of much more of the available evidence base. Current ANRs and INLs are based on datasets that are appreciably smaller than 1000 [2], whereas the IS data used here consisted of 56 datasets from 49 studies with in total 15,968 subjects [5]. The summary statistics extracted from these studies and used as input in the current work are available as File S1. In this paper we did not address the question which are exactly the populations to be modelled. More experience is needed to learn which stratifications are necessary, e.g. should national populations, age classes or sexes be modelled separately, or can they be aggregated into larger groups.

To derive an estimate of the variation in true intake, repeated 24HR data from adults in the Netherlands were used. Similar analyses could be performed for other populations to see if the variation in vitamin B-12 usual intake (SDNI) can be assumed to be equal or that stratification is necessary. Note that mean intakes (ANI) may differ between populations without influencing the results as long as the point (ANI, ANS) is assumed to lie on the same biologically determined dose-response line.

Discussion is possible about the appropriateness of cut-off values such as 150 pMol/L for plasma vitamin B-12 set by a WHO Consultation [29], [30], [31]. The need for an appropriate cut-off is shared with currently used NIVs, e.g., deriving the ANR based on the balance method used in France and The Netherlands critically depends on assumptions on liver stores necessary to maintain health [32]. Vitamin B-12 requirements set by IOM are based on achieving stable haemoglobin, normal mean cell volume and normal reticulocyte response as the health endpoints. Depletion-repletion studies are based on the same principle of achieving a specified response at the individual level. Without a cut-off for an (intermediate) health marker inference is necessarily limited to proposing an adequate intake, which is a NIV not based on requirements, but on observed intakes [10].

### Model

For estimating the slope of the intake-status linear function, we considered several models (Table 2). The results (Tables 5, 6, 7, Figure 3) clearly illustrate that this choice has a major influence on the estimated nutrient intake values, although less so for PNL_97.5_ than for ANR INL_97.5_ and MTUNID_97.5_. The purpose of the current paper is to show methodological possibilities. For real assessments of NIVs it will be necessary to assess the validity of the different assumptions that have to be made.

Using a meta-analysis of RCT data (model A) may seem the most promising because most direct way to estimate the dose-response relation. However, our results indicate some potentially disturbing facts: starting from a cut-off value of 150 pMol/L the ANR is estimated at 0.078 μg/d which is much lower than ANR values currently used (1–2 μg/d). In addition the variation in nutrient requirements is estimated to be enormous (CVNR = 1736%), and a distinct distribution-widening intake-related bias is found (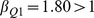), quite contrary to the distribution-narrowing effects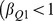) found in other studies [25]. Despite the conceptually strong status of the RCT, all these results cast some doubt on model A. The very low ANR and relatively high INL and PNL values obtained in the RCT-based model show that non-linearity may be an issue in the case of vitamin B-12 biomarkers. In particular, there may be doubt about the linearity when RCTs with relatively high doses are used because many biomarkers will have a level of saturation [33], [34]. Typical RCT data have two doses, where the low dose is in the same range as observational data, but the high dose is much higher. With only two doses it is not possible to check linearity of the dose-response relation for single datasets. If non-linearity at high doses would be considered likely then a restriction to dose levels within the linear range is advisable. If the slope is steeper indeed at lower levels of intake, then our results would shift into steeper slopes, as in models B to D.

In many existing derivations of INLs a CV of 10–20% for requirements is assumed [28]. Such values are typically based on very limited information [2]. Assuming a traditional value for variation in nutrient requirements, e.g. CVNR = 20%, as in model C, is an alternative to the RCT-based model. A remarkable fact is that model C does not require simultaneous intake-status data. This model leads indeed to a more traditional ANR estimate (1.8 μg/d), and to a distribution-narrowing intake-related bias (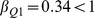). However, under this model the correlation between true intake and true status is estimated to be very high (0.92, see Figure 3C). It may be more realistic to assume that the requirements variation is larger than often assumed, thus explaining the rather low correlation found in practice. The true situation could perhaps be something between the results of model B (where there is no intake-related bias and the correlation is estimated to be 0.33) and model C. Given these uncertainties, it is reassuring that the PNL estimates, which are the prime outcomes of our method, are relatively insensitive to this model choice: PNL_97.5_ is estimated to be 6.4 and 4.8 μg/d under models B and C, respectively.

Intake-related bias in the repeated 24-hour recall method was found to be present in the OPEN study [25]. In a sensitivity analysis we showed that allowing for the order of magnitude of intake related bias in the repeated 24-hour recall data found for energy and protein in the OPEN study, the calculated PNLs changed by at most 5% in models A, C and D (model B is less relevant in this sensitivity analysis: it is strange to model intake-related bias in the 24-hour recall data but not in the IS_obs_ data, whereas the evidence for this type of bias is much stronger for the latter type of data).

The bivariate normal model (Figure 1B) is a simple approximating model. Distributions may be more complex in reality. Future distributions of intake and status, foreseen as the result of public health planning, are assumed, as in IOM [11], to be just shifted versions of the current distribution. Validation is needed for the appropriateness of the bivariate lognormal model as a fit-for-purpose approximation. Considering the scarcity of relevant and precise data in many cases, a simple model may be preferable over more complex models.

### Further research

If requirements variation is not negligible compared to intake variation, as suggested in this paper, then the MTUNID approach of IOM [11] has to be updated as we do with PNL.

Our model assumes ln-ln linear relationships, and we noted that this leads to uncommon estimates of ANR (very low), person-specific intake variance beyond the intake-related bias (zero) and CVNR (very high), possibly because of non-linearity and saturation. Other dose-response curves, e.g. S-shape curves might perform better, but have not been used in this context. We have simply assumed parallel slopes for individuals (see Figure 1A). Current thinking in biology might suggest that there is substantial variation in biological response to the same exposure, not only in terms of additive effects to the status level achieved, but possibly also in terms of the slope of the associations. It was beyond the scope of this paper and far beyond current practice to incorporate such considerations into the model.

Another possible extension of the model is to consider more dimensions than just the bivariate distribution of usual intake and one health-related variable. For example, one might consider bone mineral density as a more direct health measurement for the effects of vitamin B-12. Data on the three marginal distributions of intake, status and health plus data on the bivariate intake-status, intake-health and status-health relations can all be integrated to estimate the parameters of a trivariate lognormal model, assuming that intake would influence health only through status as an intermediate variable (conditional independence assumption). Given a cut-off value on the health parameter, we could then apply our methods to the marginal intake-health distribution, which would however be better estimated through the use of the underlying status data. We experimented with this for the vitamin B-12 case, but do not report any results here because we currently found insufficient data on the status-health relation to be able to apply the model. However, other cases may exist where such a model could be feasible.

We conclude that use of biomarker data with our extended meta-analytical approach to estimate the joint distribution of intake and biomarkers more precisely offers possibilities for setting more science-based nutrient intake values. Further refinement of methods and exploration using data on other nutrients is desirable.

## Supporting Information

Appendix S1 Derivation of nutrient intakes under the stochastic model.
(PDF)

File S1 The compressed file ‘Vitamin B12 Intake-Status summary statistics.zip’ contains two Excel tables containing the Vitamin B12 summary statistics for observational and RCT data.
(ZIP)
