# Correction: A Statistical Method to Base Nutrient Recommendations on Meta-Analysis of Intake and Health-Related Status Biomarkers

**DOI:** 10.1371/journal.pone.0106168

**Published:** 2014-08-13

**Authors:** The PLOS ONE Staff

There are multiple errors in Tables 5, 6, and 7. Please see the corrected Table 5 here.

**Table 5:** Estimated Nutrient Intake Values for four models (A-D) based on health-related cut-off value 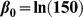.

Model	ANR (EAR)	INL_97.5_ (RDA)	PNL_97.5_	MTUNID_97.5_
	(µg/d)	(µg/d)	(µg/d)	(µg/d)
	(Eq. 2)	(Eq. 3)	(Eq. 5)	(Eq. 6)
A (RCT-based)	0.078	8.5	9.2	0.19
B (obs-based)	0.45	5.5	6.4	1.1
C (CVNR 20%)	1.8	2.7	4.8	4.4
D (max slope)	1.9	1.9	4.7	4.7
ratio A/D	0.04	4	2	0.04

Please see the corrected Table 6 here.

**Table 6:** Estimated Nutrient Intake Values for four models (A-D) based on health-related cut-off value 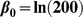.

Model	ANR (EAR)	INL_97.5_ (RDA)	PNL_97.5_	MTUNID_97.5_
	(µg/d)	(µg/d)	(µg/d)	(µg/d)
	(Eq. 2)	(Eq. 3)	(Eq. 5)	(Eq. 6)
A (RCT-based)	0.42	45	49	1.0
B (obs-based)	1.1	14	16	2.8
C (CVNR 20%)	2.6	3.8	6.7	6.2
D (max slope)	2.7	2.7	6.4	6.4
ratio A/D	0.16	17	8	0.16

Please see the corrected Table 7 here.

**Table 7:** Estimated Nutrient Intake Values for four models (A-D) based on health-related cut-off value 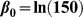
and a 25% increased SDNI

Model	ANR (EAR)	INL_97.5_ (RDA)	PNL_97.5_	MTUNID_97.5_
	(µg/d)	(µg/d)	(µg/d)	(µg/d)
	(Eq. 2)	(Eq. 3)	(Eq. 5)	(Eq. 6)
A (RCT-based)	0.078	8.1	9.2	0.24
B (obs-based)	0.13	7.1	8.2	0.40
C (CVNR 20%)	1.5	2.3	5.0	4.6
D (max slope)	1.6	1.6	4.9	4.9
ratio A/D	0.05	5	2	0.05
