# Supplementary material for: A Statistical Method to Base Nutrient Recommendations on Meta-Analysis of Intake and Health-Related Status Biomarkers
Source: PLoS One. 2014 Mar 28;9(3):e93171. doi: 10.1371/journal.pone.0093171 (PMC3969369; doi:10.1371/journal.pone.0093171)
Supplement: Appendix S1 — Derivation of nutrient intakes under the stochastic model. (PDF) [file pone.0093171.s001.pdf]

A Statistical Method to Base Nutrient Recommendations on Meta-Analysis of Intake and Health-Related Status Biomarkers

Hilko van der Voet, Waldo J. de Boer, Olga W. Souverein, Esmée L. Doets, Pieter van 't Veer

Appendix S1: Derivation of nutrient intakes under the stochastic model.

The stochastic model (bivariate normal distribution for log intake and log status) implies a linear regression line when predicting ln(status) from ln(intake). For error-free observations this relation is

$$S_i = ANS + \beta_1 \left(I_i - ANI \right) \qquad \text{(A.1)}$$

with subscript *i* indicating any individual person in the population of interest.

In our model it is assumed that for each individual *i* the measured ln status (y_i) is a linear function of the deviation of the true ln intake (I_i) from the individual ln requirement (R_i), plus random measurement error (e_i) with mean 0 and variance σ_e^2. The measured ln intake (x_i) is assumed to be also a linear function of the true ln intake (I_i), plus random measurement error (d_i) with mean 0 and variance σ_d^2. Parallel lines are assumed for all individuals. The model can thus be written as

$$y_i = S_0 + \beta_1 \left(I_i - R_i \right) + e_i ; \quad x_i = \beta_{Q0} + \beta_{Q1} I_i + d_i \qquad \text{(A.2)}$$

The parameters S_0 and β_1 are the parameters of interest in the 'disease model', where S_0 is the ln status value expected for any individual who exactly satisfies his personal intake requirement. This is the external nutrient status cut-off value to classify health as sufficient or insufficient. The parameters β_{Q0} and β_{Q1} describe the general and intake-related bias in the model describing questionnaire data used to assess nutrient intake. Note that absence of general bias at the average nutrient intake (*ANI*) level is represented by $\beta_{Q0} = \left(1 - \beta_{Q1}\right) ANI$, so that $x_i = ANI + \beta_{Q1} \left(I_i - ANI \right) + d_i$. Assuming also absence of intake-related bias we would set $\beta_{Q1} = 1$, reducing the model to the simple measurement error model $x_i = I_i + d_i$.

The relation between the observed quantities x_i and y_i is obtained by elimination of the unknown true intake. After introducing centring constants *ANI*, *ANS* and *ANR* for x, y and R respectively, the model can be written as

$$y_i = ANS + \beta_1 \beta_{Q1}^{-1} \left(x_i - d_i - ANI \right) + \beta_1 \left(ANR - R_i \right) + e_i \qquad \text{(A.3)}$$

where

$$ANR = \left(ANI - \beta_{Q0}\right) / \beta_{Q1} + \left(S_0 - ANS\right) / \beta_1 \quad \text{(A.4)}$$

In the following we assume absence of general bias in the observed intakes, in which case the latter equation simplifies to

$$ANR = ANI + \left(S_0 - ANS\right) / \beta_1 \quad \text{(A.5)}$$

ANR is therefore the intersection of the line with slope β_1 through (*ANI*, *ANS*) with the line y = S_0.

ANR and INL_p are defined as the median and p[th] percentile of the nutrient requirements distribution. INL_p is *ANR* plus an appropriate multiple of the requirements standard deviation:

$$INL_p = ANR + \text{z}_p \; SDNR \quad \text{(A.6)}$$

In the bivariate model the deviations from the true regression line are explained by variation in individual requirements around *ANR* (see Figure 1A). Consequently,

$$SDNS^2 = \beta_1^2 \left(SDNI^2 + SDNR^2\right) \quad \text{(A.7)}$$

and, given the true parameters of the bivariate distribution, the standard deviation of nutrient requirements *SDNR* and the coefficient of variation for nutrient requirements CVNR can be estimated by

$$SDNR = \sqrt{(SDNS / \beta_1)^2 - SDNI^2}\,; \qquad \text{CVNR} = \sqrt{\exp\left(SDNR^2\right) - 1} \quad \text{(A.8)}$$

The PNL is defined as the intake level where p % of the *S* distribution is above S_0, so from equation A.1

$$S_0 + z_p SDNS = ANS + \beta_1 \left(PNL_p - ANI\right) \quad \text{(A.9)}$$

which using equation 5 can be rewritten as

$$PNL_p = ANR + z_p \, SDNS / \beta_1 \quad \text{(A.10)}$$
